# SARS-CoV-2/Omicron subvariants: global outbreak upsurge and expected upcoming threats

**DOI:** 10.1097/JS9.0000000000000240

**Published:** 2023-02-16

**Authors:** Sumira Malik, Priyanka Uniyal, Jutishna Bora, Shafiul Haque, Sarya Swed

**Affiliations:** a Amity Institute of Biotechnology, Amity University Jharkhand, Ranchi, Jharkhand; b Department of Pharmaceutical Sciences and Technology, Sardar Bhagwan Singh University, Dehradun, Uttarakhand, India; c Research and Scientific Studies Unit, College of Nursing and Allied Health Sciences, Jazan University, Jazan, Saudi Arabia; d Centre of Medical and Bio-Allied Health Sciences Research, Ajman University, Ajman, United Arab Emirates; e Gilbert and Rose-Marie Chagoury School of Medicine, Lebanese American University, Beirut, Lebanon; f Faculty of Medicine, Aleppo University, Aleppo, Syria

*To the Editor:*

The Omicron (B.1.1.529) variant of severe acute respiratory syndrome coronavirus 2 (SARS-CoV-2) was previously reported through WHO on 26 November 2021 as a variant of concern due to its immense potential in escaping and masking of the immune system from the host. Likewise, a similar predisposition has been reported in its sublineages attributed to spontaneous mutations in its spike protein. The major sublineages of the Omicron variant are BA.1, BA.1.1, BA.2, BA.3, BA.4, BA.5, BA.2.75, BA.4.6, BF.7, BQ.1, and BQ.1.1^1^. The Omicron subvariant BF.7 (BA.2.75.2) is the major pathogenic causative agent accountable for surged cases in China and other countries^2^. Over time, SARS-CoV-2 has acquired several genetic mutations resulting in multiple types of SARS-CoV-2 variants and subvariants. Timely, the Omicron BA.2 surpassed BA.1, which is attributed to its subvariant’s sudden enhanced invasion and transmissibility and predisposition to re-infect previously exposed patients^3^. Following BA.2, an overabundance of its subvariants appeared, depicting rapid progression with frequently diversified circulating SARS-CoV-2 resulting in the formation of multiple subvariants such as BA.4.6, BF.7, BQ.1, BQ.1.1, BA.2.75.2, and XBB. Moreover, the prevalence of these new subvariants, especially BA.5.2.1.7, is at the peak elevation along with the original Omicron subvariant^4^. As per reported cases, Omicron subvariants alters, modifies, and prefer altered entrance pathway, which was found to be linked with their variable pathogenic approach and lung tropism^5^,^6^.

As of 19 December 2022, more than 64 million confirmed cases and 6,656,601 deaths, with 159,232 new cases in the last week were reported of coronavirus disease 2019 (COVID-19) have been reported across the world due to COVID-19^7^. As per epidemiological cases, SARS-CoV-2 has been actively transmitting around the globe over the past 2 years. During the past several weeks, Omicron BA.2 variant has been found to be the dominant strain in Denmark, India, Norway, and Singapore, indicating its ascendency over the Omicron BA.1 variant.

In January 2022, the subvariants of Omicron BA.1 and BA.2 were mainly responsible for few BA.5.2.1.7 driven infections, and four cases of BF.7 have been diagnosed in India so far. In all, 3.9% uprising cases of the BA.5.2.1.7 variant were reported since the end of December 2022 in the USA, while 7% in the UK. Globally, WHO has received reports of 651,918,402 confirmed cases of COVID-19, including 6,656,601 mortalities, on 23 December 2022^8^. China reported a total of 10,167,676 COVID-19 cases, with 31,585 confirmed deaths till 28 December 2022, with 183,316 cumulative cases and 472 deaths in the last week. In China, Omicron subvariant BF.7 (BA.2.75.2) and other countries are indicating the alarming situation

As of now, the SARS-CoV-2 Omicron variant BA.2.10.1–BA.2.75 has been reported in 35 countries, as indicated by WHO^3^. Till 19 December 2022, SARS-CoV-2, 99,950 sequences (99.7%) were globally reported as the Omicron variant of concern. In week 48 (28 November–4 December 2022), BA.5 and its sublineages are likely to be dominant at the global level as per the genomic data submission in GISAID. The prevalence of BA.2.75 accounts for 12.6% of sequences submitted. At the global level, in week 48, six variants’ BQ.1* (42.5%), BA.5 (S:R346X, S:K444X, S:V445X, S:N450D, S:N460X) with one or several of five mutations (13.4%), BA.2.75* (9.8%), XBB* (6.1%), BA.4.6* (1%), and BA.2.30.2* (0.1%) are currently placed under monitoring account of WHO for causing global threat (https://www.who.int/publications/m/item/covid-19-weekly-epidemiological-update---21-december-2022).

For SARS-CoV-2, WHO’s six regions reported the data on new ICU admissions till 21 December 2022 (week 49) from 21 (9%) countries. The region with the highest cases was the European Region (13 countries; 21%), followed by the Eastern Mediterranean Region (4 countries; 18%), the Western Pacific region (2 countries; 9%), and the African Region (1 country; 2%). No country in the South-East Asia Region or the Region of the Americas has reported the data on new ICU admissions during week 49. At the regional level, the number of newly reported weekly cases decreased across four of the six WHO regions: the South-East Asia Region (−36%), the African Region (−29%), the Eastern Mediterranean Region (−26%), and the European Region (−16%); while, the reported cases have been increased in two WHO regions: the Western Pacific Region (+8%) and the Region of the Americas (+18%) (https://www.who.int/publications/m/item/covid-19-weekly-epidemiological-update---21-december-2022).

As per the immunological data, to combat SARS-CoV-2 Omicron subvariants and especially the Omicron BA.5.2.1.7 variant, due to the immunological escape and functional alteration to the spike protein (S) in these new Omicron subvariants warrant future expedited research with dedicated funding program in amalgamation with governmental and private entities involving multicentre/collaborative research programs. In conclusion, the individuals vaccinated with the triple vaccine against Omicron BA.1 and BA.5 showed increased neutralization resistance in the sera upon the infection from BA.5.2.1.7, majorly due to N460K mutation. Further, XBB.1 and XBB.3 variants have been reported as more immunoevasive than BA.5.2, and the patients infected with BA.5.2 failed to elicit neutralizing antibodies against the XBB sublineage. Thus, patients infected with BA.5 may face a higher risk of symptomatic reinfection from the XBB sublineage than their previous sublineages (https://www.who.int/publications/m/item/covid-19-weekly-epidemiological-update---21-december-2022)^9^.

The current correspondence focuses on the upcoming threats due to consistent and rapidly evolving SARS-CoV-2 and Omicron variants with enhanced immune evasion affecting public health at the global level. This warrants novel modalities for urgent monitoring of immunological evasion of developing variants and creating a deeper understanding of accountable factors associated with the increased viral transmissibility. The suggested approach might serve as a critical factor for improving the reformulation of mRNA vaccines and aid in analyzing novel wider coronavirus vaccine candidates toward effective prevention strategies.

## Ethical approval

The authors declare no animal studies or human participants involvement in the study as it is a compiled letter article.

## Sources of funding

No funding was received.

## Author contribution

S.M. and P.U.: designed the study; S.M., P.U., and S.K.: made the first draft; S.M., P.U., and S.K.: updated the manuscript; S.M. and K.D.: reviewed the final draft. All authors have critically reviewed and approved the final draft and are responsible for the content and similarity index of the manuscript.

## Conflicts of interest disclosure

There are no conflicts of interest.

## Research registration unique identifying number (UIN)

1. Name of the registry: NA.

2. Unique identifying number or registration ID: NA.

3. Hyperlink to your specific registration (must be publicly accessible and will be checked): NA.

## Guarantor

Dr Sumira Malik and Sarya Swed.

## Data availability

Data not available/not applicable.
